# Aristaless Related Homeobox (ARX) Interacts with β-Catenin, BCL9, and P300 to Regulate Canonical Wnt Signaling

**DOI:** 10.1371/journal.pone.0170282

**Published:** 2017-01-19

**Authors:** Il-Taeg Cho, Youngshin Lim, Jeffrey A. Golden, Ginam Cho

**Affiliations:** Department of Pathology, Brigham and Women’s Hospital, Harvard Medical School Boston, Massachusetts, United States of America; Hopital Robert Debre, FRANCE

## Abstract

Mutations in the *Aristaless Related Homeobox* (*ARX*) gene are associated with a spectrum of structural (lissencephaly) and functional (epilepsy and intellectual disabilities) neurodevelopmental disorders. How mutations in this single transcription factor can result in such a broad range of phenotypes remains poorly understood. We hypothesized that ARX functions through distinct interactions with specific transcription factors/cofactors to regulate unique target genes in different cell types. To identify ARX interacting proteins, we performed an unbiased proteomics screen and identified several components of the Wnt/β-catenin signaling pathway, including β-catenin (CTNNB1), B-cell CLL/lymphoma 9 (BCL9) and leucine rich repeat flightless interacting protein 2 (LRRFIP2), in cortical progenitor cells. Our data show that ARX positively regulates Wnt/ β-catenin signaling and that the C-terminal domain of ARX interacts with the armadillo repeats in β-catenin to promote Wnt/β-catenin signaling. In addition, we found BCL9 and P300 also interact with ARX to modulate Wnt/β-catenin signaling. These data provide new insights into how ARX can uniquely regulate cortical neurogenesis, and connect the function of ARX with Wnt/β-catenin signaling.

## Introduction

The Aristaless related homeobox (ARX) protein, the vertebrate homolog of Drosophila Aristaless, is a paired-like transcription factor with temporally and spatially restricted expression in the developing central nervous system [1]. Mutations in *ARX* result in a broad range of neurologic phenotypes from severe structural anomalies of the brain with accompanying epilepsy and intellectual disabilities, to structurally normal brains having similar intellectual disabilities and epilepsy [2,3]. Furthermore, there is a reasonably close correlation between the type of mutation in *ARX* and the associated phenotype [4].

During brain development *Arx* is expressed in neural progenitor cells (NPC) located in both the ventricular zone (VZ) of the pallium (neocortex) and the subventricular zone (SVZ) of the subpallium (ganglionic eminence, GE)[1,5]. Postmitotic cortical projection neurons derived from the pallial VZ down-regulate *Arx* expression as they exit the cell cycle and migrate to the cortical plate. In contrast, interneurons from the subpallial SVZ continue to express ARX during their non-radial migration and expression persists in mature post-migratory interneurons [5]. Of considerable interest, ARX appears to play independent roles in these two progenitor populations and yet another role in the development of the ventral spinal cord [6–10]. We hypothesized that a distinct repertoire of ARX interaction partners in different cell types confer context-dependent transactivational activities to ARX [7–11]. Thus, identifying the components of the functional complexes in each progenitor population will provide mechanistic understandings as to how one transcription factor can independently regulate distinct cellular functions in different NPCs.

In the current study, we took an unbiased proteomics approach to identify ARX interacting proteins in the embryonic mouse forebrain. We identified multiple components of the Wnt/β-catenin (canonical) signaling pathway expressed in the cortical (pallial) VZ, including β-catenin (CTNNB1), LRRFIP2, and BCL9. This pathway is known to function in NPC proliferation and differentiation, intermediate progenitor cell (IPC) generation, and cortical lamination [12]. We found that ARX positively regulates Wnt/β-catenin signaling through interactions with β-catenin, BCL9 and P300. These data provide new insights into how ARX functions in cortical neurogenesis and how a single transcription factor can have distinct functions depending on cellular context resulting in specific patient phenotypes.

## Materials and Methods

### Animals, In utero electroporation (IUEP), and immunofluorescent imaging

All mouse studies were performed in accordance with the guidelines in the Guide for the Care and Use of Laboratory Animals of the National Institutes of Health. The experiments were approved by Harvard Medical School’s Institutional Animal Care and Use Committee (*protocol number*: *04946*). *CD1* mice were purchased from Charles River Laboratories. *In utero* electroporation (IUEP) was performed as described previously on embryonic day 12.5 (E12.5) [11]. One day after IUEP, the dams were sacrificed and the brains were removed. The E13.5 embryonic brains were fixed overnight in 4% paraformaldehyde and prepared for cryosectioning (15 μm) as described [7]. All sections were imaged using Zeiss Zen Pro software driving a Hamamatsu ORCA-Flash 4.0 camera attached to a Zeiss Observer Z1 inverted microscope (Carl Zeiss, Oberkochen, Germany).

### Plasmids

*Arx* truncation and amino acid substitution mutants were made by site-directed mutagenesis. Briefly, primers were designed using Agilent QuikChange Primer Design software and PCR was performed by KOD Xtreme^™^ Hot Start DNA Polymerase (Novagen) using pCAG-Arx-IRES-GFP as a template [7]. The PCR products were digested with DpnI (NEB) and transformed into DH10B *E*. *coli*. Primers used in this study are listed in the supplemental table (S1 Table). Full-length β*-catenin* and *ΔN89-*β*-catenin* (N-terminal deletion of β-catenin) [13] were amplified using a mouse brain cDNA (E12.5) and cloned into pCIG or pEBG vector using GeneArt cloning method (Invitrogen). pM-β-catenin was constructed by ligating β*-catenin* (MfeI/XbaI) into the pM (EcoRI/XbaI) vector for GAL4DB fusion protein expression. *ΔN89-*β*-cateninΔC* and *ΔN89-*β*-catenin D164A* mutants were made by site-directed mutagenesis as described above. pcDNA3-based constructs (*pcDNA3-Arx-FLAG*, *pcDNA3-HA-Lrrfip2*, *pcDNA3-HA-Bcl9*) were cloned using the pcDNA3.1 Directional TOPO Expression kit (Invitrogen). Amino acid substitution mutants in NLS2 (327–334, **KRK**QRRYR→ **AAA**QRRYR), NLS3 (381–388, **RR**A**K**WR**K**R→**AA**A**A**WR**A**R), or aristaless domain (529–534, **RR**A**SSI** →AAAAAA) were made by site-directed mutagenesis. DNA constructs used for IUEP include: *pTOP-dGFP-NLS-CAG-DsRed*, *pCAG-Arx* or *pCAG*. The oligonucleotides used in this study are described in S1 Table. All Plasmid constructs were confirmed by sequencing (Macrogen, USA).

### Cell culture and transfection

HEK293T cells were cultured in Dulbecco’s modified Eagle’s medium GlutaMAX (Invitrogen, Life Technologies, Grand Island, NY) containing 10% fetal bovine serum. Transfection was performed using PEI reagent (Polysciences, PA) as described [14].

### Mass spectrometry

The whole embryonic forebrains (E14.5) were triturated in buffer B (25 mM Tris-Cl, pH 7.4, 5% glycerol, 3 mM KCl, 140 mM NaCl, 1% Triton X-100, 0.2 mM EDTA, 1.5 mM MgCl_2_, protease and phosphatase inhibitors; Roche Biochem), and sonicated on ice (Branson digital sonifier 250, 5 cycles of sonication at 2 sec “On” and 2 sec “Off’ at 10% output). The crude lysates were centrifuged at 13,000 x g for 15 min at 4°C, and the supernatants were pre-cleared with protein A/G agarose bead for 1 hr at 4°C with rotation. In order to isolate novel ARX-binding proteins, ARX antibody against amino acids 158–181 was generated in rabbit and immobilized on Dynabeads M-280 Tosylactivaed (Invitrogen) according to the manufacturer’s instructions, and the beads were incubated with the pre-cleared whole embryonic brain lysate for 2 hr at 4°C with rotation. The beads were washed in buffer B three times (each time for 3 min at 4°C with rotation), and bound protein were acid-eluted and immediately neutralized. The eluted proteins were analyzed at the Keck Mass Spec & Proteomics Resource (Yale School of Medicine) and Proteomics & Systems Biology core (Perelman School of Medicine at the University of Pennsylvania).

### GST-pull down assay and immunoprecipitation

GST pull-down assays were performed as described previously [15]. After transfection of GST fusion expression constructs, cells were lysed in TNE buffer (20 mM Tris-HCl, pH 7.4, 150 mM NaCl, 0.5% Triton X-100, 5% glycerol) containing a protease inhibitor mixture (Roche Biochem). After centrifugation, supernatants were mixed with glutathione-sepharose 4B (GE Healthcare Bio-Science AB) and incubated for 45 min on ice. The unbound proteins were next washed with TNE buffer five times and the eluates were analyzed by Western blot.

For immunoprecipitation, cells were transfected and then lysed 1 day later in TNE buffer (20 mM Tris-HCl [pH 7.4], 150 mM NaCl, 0.5% Triton X-100, 5% glycerol) containing a protease inhibitor cocktail (Roche Biochem). For preclearance, lysates were incubated with protein G-conjugated beads (Invitrogen). Two micrograms of primary antibody (anti-ARX) were incubated at 4°C for 2 h, and then incubated with the protein G-conjugated beads for an additional hour. The beads were washed with TNE buffer twice and then two more times with TNE buffer containing 500 mM NaCl, followed by a final wash with TNE buffer. Bound proteins were eluted using sodium dodecyl sulfate-polyacrylamide gel electrophoresis (SDS-PAGE) sample buffer. To detect the associated proteins in the immunoprecipitates, SDS-PAGE followed by Western blotting was performed. Anti-ARX (1:200, generated by the polypeptide in rabbit) [6], anti-FLAG (M2, 1:500; Sigma), anti-CBP (A-22, 1:1,000, Santa Cruz), anti-MYC (9E10, 1:100; Sigma), anti-HA (C29F4, 1:500, Cell Signaling Technology), anti-CTNNB1 (1:1,000, Chemicon), anti-BCL9 (1:1000, Abcam), anti-LRRFIP2(1:1000, Santa Cruz) antibodies were used for immunoblotting.

### Luciferase assay

**Fig 1:**
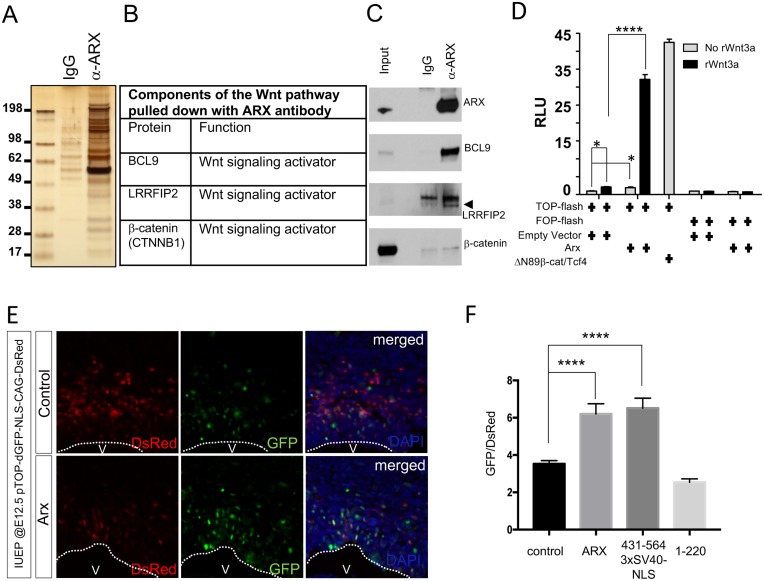
ARX interacts with multiple components of the canonical Wnt signaling and activates this pathway. (A) Identification of ARX-binding proteins. Whole brain lysates of the E14.5 mice were used for immunoprecipitation with IgG (control) or an ARX antibody and the resulting proteins were subjected to SDS-PAGE and silver-stained. (B) Co-eluted proteins were analyzed by tandem mass-spectrometry (MS-MS). Among the proteins identified were several components of the Wnt/β-catenin signaling pathway. (C) Confirmation of binding between ARX and candidate molecules by Western blot using whole brain lysates (E14.5) immunoprecipitated with ARX antibody. Immunoprecipitates were probed with the indicated antibodies (note: although the β-catenin background is high, the immunoprecipitated band was consistently stronger than the IgG control). (D) Quantification of the TOP-flash or FOP-flash (mutant TCF binding site) reporter assay in HEK293T cells transfected with empty vector or *Arx* expression construct (100ng), and treated with or without rWnt3a (100ng/ml, at which concentration Wnt3a induces minimal level of Wnt signaling activity). *Δ*N89-β-cat/TCF (fusion construct of stabilized β-catenin and TCF) was used as a positive control. RLU, relative light unit; rWnt3a, recombinant Wnt3a. ****p<0.*0001* and *p<0.*05* (n = 3). (E) Representative images of embryonic cortical sections electroporated with pTOP-dGFP-NLS-CAG-DsRed (Wnt/β-catenin reporter construct), together with control or *Arx* expression construct (EP at E12.5 and harvested at E13.5). GFP expression indicates the level of canonical Wnt signaling activity. DsRed expression is an internal control of the electroporated cells. Dotted lines outline ventricle side of neocortex. V; ventricle. (F) Quantification of the Wnt reporter activities in the E13.5 cortical sections expressing control (*pCAG*), *Arx (WT)*, *Arx (431-564-3XSV40-NLS)*, or *Arx (1–220)*, together with reporter construct (pTOP-dGFP-NLS-CAG-DsRed), after IUEP at E12.5. The ratio of green intensity to red intensity (GFP/DsRed) was calculated from DsRed-positive cells located only at the VZ of the cortical sections electroporated with the indicated constructs (****p<0.*0001*, n = 65 cells from at least 10 sections of 2–4 embryos for each construct).

**Fig 2:**
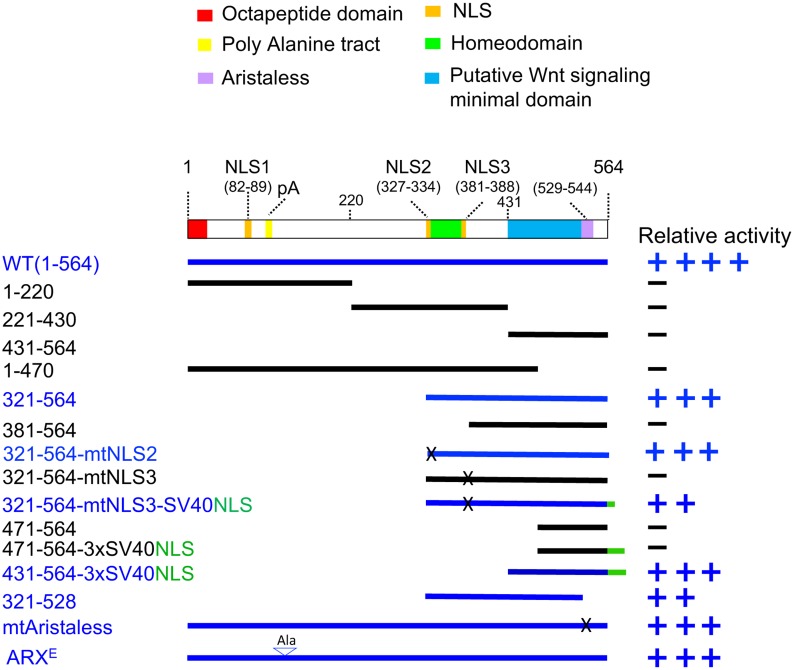
C-terminal domain of the ARX is crucial for Wnt/β-catenin upregulation by ARX. A schematic representation of ARX with functional domains highlighted (Top). Wild type and mutant *Arx* constructs (left) are presented as black (inactive) or blue (active) lines according to their transcriptional activation activity in the TOP-flash reporter assay (HEK293T cells). The levels of Wnt/β-catenin signaling activation by these different ARX derivatives are indicated on the right. The values are presented as follows: ++++, 75–100% stimulation, considered equivalent to WT ARX; +++, 50–75% stimulation; ++, 25–50% stimulation; +, 10–25% stimulation; -, less than 10% stimulation, equal to the reporter alone activity upon rWnt3a treatment. Abbreviations: NLS, nuclear localization signal; mt, mutated; ARX^E^, *Arx* mutant with first poly alanine tract expansion. mtAristaless has multiple point mutations at the conserved amino acids, R529, R530, S532, S533 and I534.

**Fig 3:**
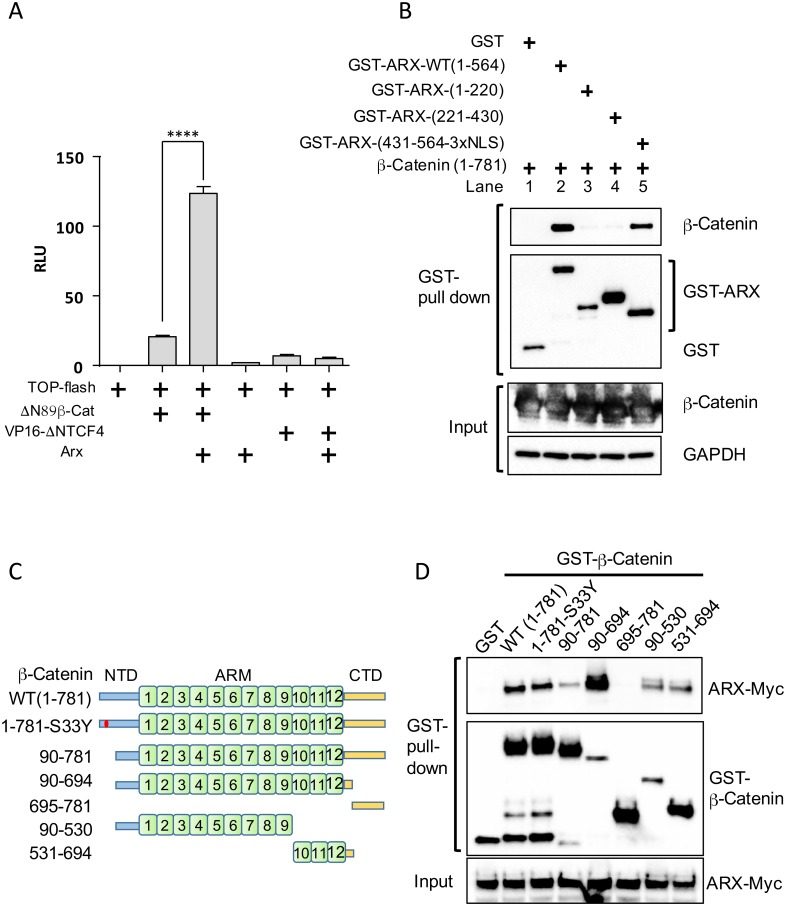
ARX activates canonical Wnt signaling through its interaction with β-catenin. (A) Quantification of Wnt/β-catenin activity with TOP-flash reporter assay in HEK293T cells transfected with the indicated DNAs. *Δ*N89-β-catenin, a constitutively active form of β-catenin; Vp16-*Δ*NTCF4, a fusion protein of the VP16 activation domain with the TCF4 DNA binding domain. ****p<0.*0001* (n = 3). RLU represents relative light unit. (B) GST-pull down assay with HEK293T cells co-transfected with the indicated GST-ARX derivatives and β-catenin expression construct. (C) Schematic diagram of β-catenin derivatives used for interaction mapping. NTD, N-terminal domain (1–89); ARM, armadillo domain (90–693); CTD, C-terminal domain (694–781). S33Y mutant is another form of stabilized β-catenin (constitutively active). (D) GST-pull down assay with HEK293T cells co-expressing GST-β-catenin derivatives and ARX-MYC.

**Fig 4:**
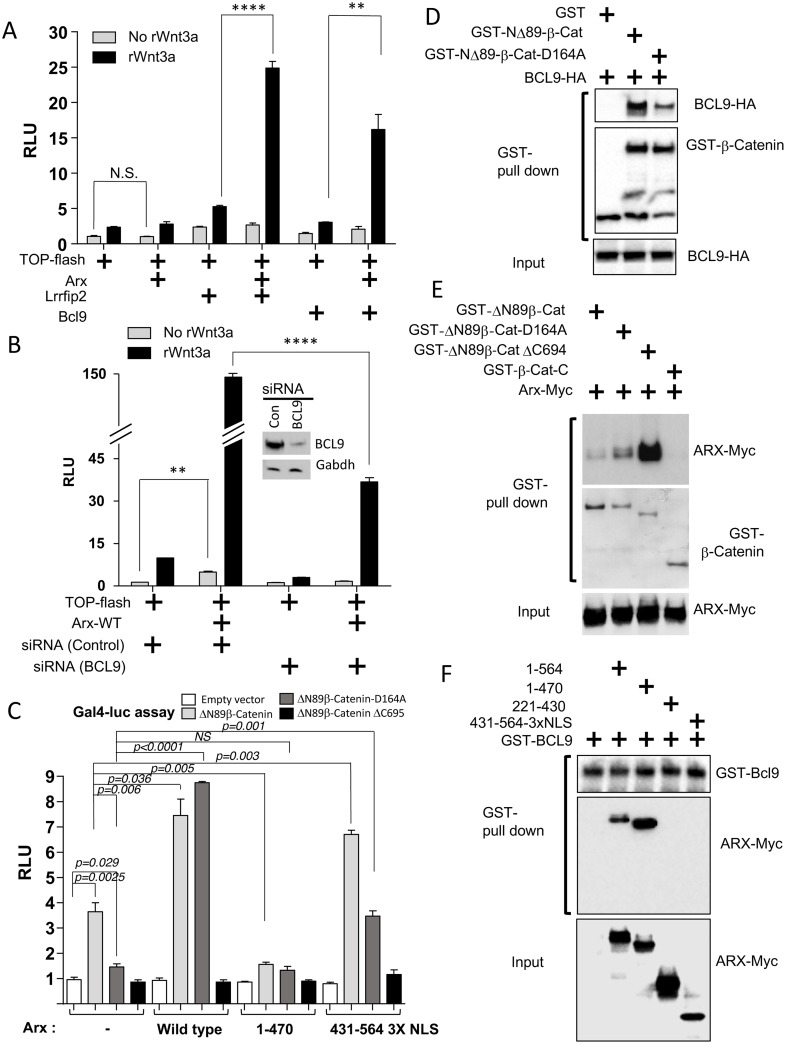
ARX cooperates with BCL9 in transcriptional co-activation of canonical Wnt signaling. (A) Quantification of TOP-flash reporter assay in HEK293T cells transfected with indicated DNAs. As described in the Methods and Materials, *Arx* expression construct was used at a low concentration that does not induce Wnt/β-catenin activity in order to maximize measurement of synergism. ****p<0.*0001*, **p = 0.*0036* (n = 3) and N.S.; Not Significant. (B) Quantification of TOP-flash reporter assay in HEK293T cells transfected with indicated DNAs or siRNAs. The Western blot above the graph shows efficient knock down of BCL9. ****p<0.*0001* and ** *p<0*.*01* (n = 3). (C) Quantification of the Gal4-luc reporter assay in HEK293 cells co-transfected with Gal4 activator, reporter, and *Arx* constructs as indicated. Three different Gal4 activators (Gal4DBD-*Δ*N89-β-catenin, Gal4DBD-*Δ*N89-β-catenin D164A, Gal4DBD-*Δ*N89-β-catenin *Δ*C695) were assayed (n = 3 for each). RLU; relative light unit. NS; non-significant. (D-F) GST-pull down assay using HEK293 cells co-expressing GST-β-catenin derivatives and BCL9-HA (D) or ARX-MYC (E), or GST-BCL9 and ARX-MYC derivatives (F).

**Fig 5:**
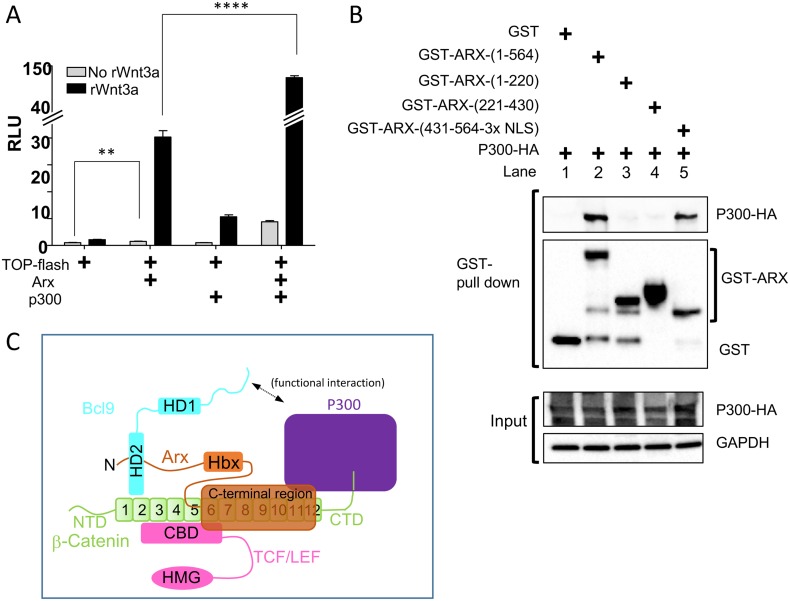
ARX cooperates with P300 to positively modulate canonical Wnt signaling. (A) Quantification of TOP-flash reporter assay in HEK293T cells expressing the indicated DNAs. RLU; relative light unit. ****p<0.*0001* and ** *p<0*.*01* (n = 3). (B) GST-pull down assay using cells co-expressing GST-ARX derivatives and P300-HA. (C) A schematic diagram summarizing the molecular interactions among ARX, β-catenin, BCL9, and P300. Interaction domain of BCL9 to ARX is arbitrary. Abbreviations: Hbx, homeodomain; HD1 and 2, homology domain; NTD, N-terminal domain; CTD, C-terminal domain; HMG, high mobility group; CBD, β-catenin binding domain.

For the TOP-flash reporter assay, HEK293T cells were plated (4 x 10^5^/well) in 12-well culture plate the day before transfection. Cells were transfected with 400 ng of luciferase reporter plasmid, 50 ng of pRL-TK Renilla luciferase as an internal control, and 100 ng (see Figs 1D, 2, 3A, 4B and 5A) or 25ng (see Fig 4A) of an *Arx* expression construct or one of the *Arx* mutant constructs using PEI (Polysciences, PA). The cells were split into poly-D-lysine coated (Sigma) 96-well plate (4 x 10^4^/well) 6 hr post-transfection and incubated for an additional 16 hr. The cells were then treated with recombinant Wnt3a (R&D system, MN) at 100 ng/ml for 8 hr, and luciferase activity measured by Dual-Glo Luciferase Assay System (Promega, CA) and POLARstar Omega microplate reader (BMG LABTECH, NC).

For silencing BCL9 in reporter assays, ON-TARGETplus Human BCL9 (607) siRNA (GE Healthcare Lifesciences) was transfected into HEK293T cells using Lipofectamin 2000 (Invitrogen). Cells were then resuspended in buffer A (25 mM Tris-Cl, pH 7.4, 10% glycerol, 150 mM NaCl, 1% Triton X-100, 0.5% sodium deoxycholic acid, 0.1% SDS,1 mM DTT, 1 mM EDTA, protease and phosphatase inhibitors; Roche Biochem) and sonicated on ice (Branson digital sonifier 250, 5 cycles of sonication at 2 sec “On” and 2 sec “Off’ at 10% output). The lysates were spun at 13,000 x g for 15 min at 4°C. Protein concentration was measured by BCA protein assay kit (Bio-Rad) and proteins were subjected to SDS-PAGE.

### Statistical analysis

All statistical analyses were done in Prism software using 2-tailed unpaired Student’s t-test. All graphs are plotted as mean ± the standard error of the mean (SEM).

## Results

### ARX interacts with components of the Wnt signaling pathway

To identify ARX interacting proteins, we performed tandem mass spectrometry (MS-MS) on protein complexes obtained from embryonic day 14.5 (E14.5) mouse forebrain that were immunoprecipitated with an anti-ARX antibody (Fig 1A). Among the proteins identified were multiple members of the canonical Wnt signaling pathway, including β-catenin (CTNNB1), leucine rich repeat flightless interacting protein 2 (LRRFIP2), and B-cell CLL/lymphoma 9 (BCL9) (Fig 1B and S2 Table). To confirm these interactions, a second immunoprecipitation was performed using E14.5 forebrain lysates and the precipitates were analyzed by Western blotting with β-catenin, LRRFIP2, and BCL9 antibodies. Our data confirmed that ARX interacts with these three proteins (Fig 1C). Finally, mRNA expression, available from public databases, confirmed that *Arx* expression overlaps with that of *Lrrfip2*, *Bcl9* and β*-catenin* in NPCs in the cortical VZ/SVZ (S1 Fig).

### ARX positively regulates canonical Wnt signaling

In canonical Wnt signaling pathway Wnt ligands bind Frizzled (Fzd)/low-density lipoprotein receptor-related protein (LRP) complex. This binding on the cell surface aggregates Dishevelled (Dsh) and functionally blocks the action of β-catenin degradation that normally occurs through the GSK3β-Axin-APC-CKIα destruction complex. The resulting accumulated β-catenin translocates to the nucleus where it interacts with T-cell factor (TCF)/lymphoid-enhancer factor (LEF) family member, bind to the promoters of the Wnt target genes (e.g. CyclinD1, Myc and Axin2), and modulate their expressions. The β-catenin/(TCF/LEF) transcription factor complex recruits several transcription co-factor proteins including BCL9/PYGO and P300/CBP, along with histone modification enzymes [16].

Given that we identified multiple members of the Wnt/β-catenin signaling pathway as ARX interacting proteins, and that their gene expression patterns overlap with *Arx* in the dorsal forebrain, we postulated ARX would participate in canonical Wnt signaling. To test this, we transfected a canonical Wnt reporter construct containing TCF/LEF binding sites (TOP-flash) or mutated TCF/LEF binding sites (FOP-flash) that drives the luciferase expression [17], with an empty vector or *Arx* into HEK293T cells. The TOP-flash or FOP-flash reporter activity was assayed in the transfected cells that were treated with or without Wnt3a. Wnt3a was used as a ligand to induce canonical Wnt signaling since it is known to function in HEK293T cells [18]. ARX, in the presence of a low concentration of Wnt3a (100ng/ml, a concentration in which Wnt3a shows minimal ability to activate canonical signaling, S2 Fig), significantly up-regulated Wnt/β-catenin signaling activity when compared to the control vector (Fig 1D and S2 Fig). However, ARX failed to induce FOP-flash activity even in the presence of Wnt3a, confirming the specificity of this up-regulation (Fig 1D). As expected, *Δ*N89-β-cat/TCF (a fusion construct of stabilized β-catenin and TCF, known to constitutively activate Wnt/β-catenin signaling, functioned as a positive control) up-regulated TOP-flash activity in the absence of Wnt3a (Fig 1D).

To confirm these *in vitro* data, we next investigated whether ARX could activate Wnt/β-catenin signaling *in vivo*. For this, a Wnt/β-catenin reporter construct, pTOP-dGFP-NLS-CAG-DsRed was introduced into the developing cortical progenitor zone using *in utero* electroporation (IUEP). This construct contains a TCF binding site that drives nuclear destabilized GFP protein (due to a short half-life) expression in a Wnt/β-catenin signaling-dependent fashion, and expresses DsRed under the chick β-actin promoter as an internal control. We co-electroporated, with this reporter construct, an empty vector *(pCAG*, *control)*, *Arx (WT)*, *Arx (431-564-3XSV40-NLS) or Arx (1–220) construct*, into the E12.5 embryonic mouse forebrain. Brains harvested at E13.5 showed that ARX strongly up-regulated GFP expression (reporter for the Wnt/β-catenin signaling activation) when compared to the control (Fig 1E). These data support our model by confirming that ARX enhances Wnt/β-catenin signaling *in vivo*. We quantified the ratio of the GFP intensity to DsRed (internal control) in each DsRed positive cell. ARX (WT) or ARX (431-564-3XSV4-NLS) showed higher GFP/DsRed ratio than that of the control or ARX (1–220). These results are consistent with our *in vitro* Wnt/β-catenin signaling reporter assay in HEK293T cells (see Fig 2), where ARX (431-564-3XSV40-NLS) exhibits comparable level of upregulating activity to that of the ARX (WT), while ARX (1–220) shows essentially no activity (see Fig 2). Together, these data further indicate Wnt/β-catenin signaling can be upregulated by ARX both in the embryonic cortex as well as in HEK293T cells, and that the C-terminal domain (431–564) of ARX is crucial for this function.

### The C-terminal domain of ARX is required to up-regulate Wnt/β-catenin signaling

ARX has multiple well defined domains including a paired-like homeobox domain conferring DNA binding specificity, N-terminal octapeptide motif repressing transcription by cooperating with TLE1, and C-terminal aristaless domain with putative transcription activation activity (Fig 2) [18]. To determine the functional domains of the ARX required for Wnt/β-catenin signaling, we generated a series of *Arx* deletion constructs and tested their capacity to function in the TOP-flash reporter assay in HEK293T cells. ARX was initially broken into three regions; N-terminal region (1–220 amino acids; aa); mid-portion of the protein (221-430aa); and C-terminal region (431-564aa). Surprisingly, none of these induced TOP-flash reporter activity (Fig 2). Thus, two larger ARX deletion mutants, ARX (1-470aa) and ARX (321-564aa), were generated, both containing the homeodomain. While ARX (1-470aa) showed marginal effect on the reporter activity, ARX (321-564aa) demonstrated comparable reporter activity to the full-length protein (Fig 2).

Given that ARX is a transcription factor, we next tested whether nuclear localization is necessary to upregulate Wnt/β-catenin signaling. The ARX (321–564) construct contains nuclear localization sequences 2 and 3 (NLS2 and NLS3) (Fig 2); we found that mutations in NLS3 abolished its nuclear localization, despite the presence of NLS2 (S3 Fig). The ARX (321–564) with the NLS3 mutation (321-564-mtNLS3) was not able to induce Wnt/β-catenin signaling, whereas the NLS2 mutant (321-564-mtNLS2) has similar activity to ARX (321–564) (Fig 2). When the ARX (321-564-mtNLS3) was fused with an SV40 NLS sequence (321-564-mtNLS3-SV40-NLS), not only was the nuclear localization restored (S3 Fig), but its capacity to activate Wnt/β-catenin signaling also returned (Fig 2). These data indicate that ARX localization within the nucleus is necessary for modulating Wnt/β-catenin signaling. These data also led us to re-examine the possibility that the defect in Wnt/β-catenin signaling activation by the ARX (431–564) mutant was due to its failure to access to the nucleus. As predicted, ARX (431–564) does not localize in the nucleus (S3 Fig). However, when three tandem SV40-NLS sequences were added (ARX 431-564-3XSV40-NLS), Wnt/β-catenin signaling activation and nuclear localization were restored (Fig 2 and S3 Fig). In the case of ARX (321–528), which lacks the aristaless domain, and ARX (mtArtistaless), which harbors multiple point mutations in the aristaless domain, retained the activation activity, implying that aristaless domain is not necessary to induce Wnt/β-catenin activity. Therefore, these data, together with those of ARX (431-564-3XSV40-NLS), imply that the 431–528 aa domain of the ARX, when localized in the nucleus, may be the putative minimal domain for induction of canonical Wnt signaling (Fig 2). In addition, the poly-alanine tract expansion mutant (ARX^E^) showed a similar level of activity to WT (Fig 2).

### Regulation of Wnt/β-catenin signaling requires physical interactions of ARX with β-catenin

We next considered how ARX promotes Wnt/β-catenin signaling activity. Given that ARX is localized in the nucleus and this nuclear localization is essential for regulating Wnt/β-catenin signaling, we focused on known components of the transcription machinery of this signaling pathway. We first examined whether ARX affects Wnt/β-catenin reporter activity through β-catenin or through TCF4 (TCF7L2), one of the TCF/LEF family members well known to function in HEK293T cells [19]. We measured the ability of ARX to induce Wnt/β-catenin reporter activity in the presence of *Δ*N89-β-catenin or Vp16-*Δ*NTCF4. *Δ*N89-β-catenin is a constitutively active form of β-catenin, which is a stabilized form of β-catenin as it lacks the GSK3β phosphorylation site which targets β-catenin for degradation [20]. Vp16-*Δ*NTCF4 is a fusion protein of the transcriptional activator, VP16, and N-terminally deleted TCF4, *Δ*NTCF4, that lacks the β-catenin interaction domain, thus containing only DNA binding domain of the TCF4 [13]; Vp16-*Δ*NTCF4 is known to induce pTOP-flash reporter activity [20]. As shown in Fig 3A, ARX enhanced *Δ*N89-β-catenin mediated Wnt/β-catenin signaling reporter activity, whereas it did not alter Vp16-*Δ*NTCF4 mediated activity. These data indicate that ARX functions through β-catenin but not directly through TCF4, suggesting the physical interaction between ARX and β-catenin results in a functional association.

To refine our understanding of the interaction between ARX and β-catenin, we studied a series of ARX truncation mutants using GST-pull down assays (Fig 3B). The 431–564 region of ARX was found to have the highest affinity for β-catenin among the three truncation constructs (Fig 3B), consistent with the functional mapping of the ARX domains in our TOP-flash assay (Fig 2). These data indicate that the C-terminal region (431–564) of ARX is required for activation of Wnt/β-catenin signaling. The inverse approach was taken to establish the binding domain of β-catenin critical for interaction with ARX. The GST-pull down assay revealed that the N-terminal region of β-catenin supports ARX binding (compare the ARX pulled down with 1–781 versus 90–781 regions of the β-catenin), whereas the C-terminal region of β-catenin inhibited ARX binding (Fig 3C and 3D). The strongest binding of ARX was achieved with the 90–694 aa region of β-catenin, suggesting that the armadillo repeats of β-catenin physically interact with ARX (Fig 3C and 3D). Taken together, amino acids 431–564 domain of ARX and the armadillo repeats of the β-catenin are crucial for their reciprocal interactions.

### ARX cooperates with BCL9 and P300 to modulate Wnt/β-catenin signaling

β-catenin is known to bind to multiple co-activators for its transcriptional activation (or transactivation) activity [16]. Given that BCL9 and LRRFIP2 are known as co-activators [21,22] and they were identified as ARX interacting proteins in our biochemical screen, we next tested whether ARX might cooperate with BCL9 and/or LRRFIP2 to enhance Wnt/β-catenin activity. TOP-flash reporter activity was measured in the presence of both proteins upon exogenous Wnt stimulation. Luciferase expression was synergistically elevated when ARX was expressed with BCL9 and also with LRRFIP2, suggesting that ARX functionally interact with both BCL9 and LRRFIP2 (Fig 4A). Note that *Arx* expression construct was used at a low concentration that does not induce Wnt/β-catenin activity in order to maximize measurement of synergism. Given that the molecular mechanism by which BCL9 enhances Wnt/β-catenin signaling is better understood than that of LRRFIP2, we focused on BCL9. To establish the necessity of *Bcl9* expression for ARX-mediated Wnt/β-catenin signal upregulation, the TOP-flash reporter assay was performed in the presence of a *BCL9* siRNA or control siRNA. Knock down of *BCL9* significantly reduced the level of luciferase activity, indicating BCL9 is required, at least in part, for ARX to promote Wnt/β-catenin signaling activity (Fig 4B).

To further delineate the relationship between ARX and BCL9 for Wnt/β-catenin signaling regulation, we took advantage of a β-catenin mutant that was previously shown to be deficient for BCL9-binding (*Δ*N89-β-catenin-D164A) [23]. The GAL4 activator/reporter system was used to measure the effect of *Arx* and its deletion constructs on transactivation activity of the three β-catenin constructs. For the GAL4 activator, we fused the DNA binding domain of the yeast Gal4 (Gal4DBD) to each of the three β-catenin mutants: 1) *Δ*N89-β-catenin, 2) *Δ*N89-β-catenin-D164A (D164A, mutation in BCL9 binding site), and 3) *Δ*N89-β-catenin *Δ*C695 (*Δ*C695, C-terminal deletion which results in no or little transcriptional activity). For the GAL4 reporter, five copies of the Gal4 binding sequences were used for the minimal promoter that drives firefly luciferase expression. Each Gal4 activator construct was co-transfected with a GAL4 reporter (Gal4-luc) construct, and one of the following *Arx* expression constructs: mock DNA, *Arx (WT)*, *Arx (1–470)*, or *Arx (431-564-3XSV40-NLS)*. The results of the Gal4-luc reporter assay, a readout of the β-catenin transactivation activity, revealed that *Δ*N89-β-catenin can function as a transcriptional activator on its own, as expected (Fig 4C; see the second bar in the first group without *Arx*). Wildtype ARX or nuclear localized C-terminal ARX (431-564-3XSV40-NLS), but not ARX (1–470), promoted the activity of *Δ*N89**-**β**-**catenin transactivation (Fig 4C), consistent with our TOP-flash reporter assay showing that nuclear localized ARX (431–564) is sufficient for the Wnt/β-catenin signaling upregulation (Fig 2). For *Δ*N89-β-catenin-D164A, the basal level of transactivation activity is significantly reduced to levels near that of *Δ*N89-β-catenin *Δ*C695 in the absence of *Arx*, or in the presence of *Arx (1–470)*. However, its activity was recovered by co-transfection of *Arx (WT)* or, to a lesser extent, *Arx (431-564-3XSV40-NLS)* (Fig 4C). We postulated that ARX might be compensating for the defective BCL9 binding to β-catenin. Interestingly, when we investigated the interaction between BCL9 and β-catenin, BCL9 maintained a weak interaction with *Δ*N89-β-catenin-D164A (Fig 4D). Furthermore, in GST-pull down assays, ARX showed a greater interaction to *Δ*N89-β-catenin-D164A than to *Δ*N89-β-catenin (Fig 4E). These results suggest that higher interaction affinity of the ARX to mutant β-catenin (D164A) might account for promotion of transactivational activity by ARX in the situation where BCL9 interaction with mutant β-catenin (D164A) is too weak to activate the pathway.

We next mapped the interaction between BCL9 and ARX. Only full-length ARX and ARX (1–470) appeared to interact with GST-BCL9. Neither ARX (221–430) or ARX (431-564-3XSV40-NLS) showed an interaction, implying that the N-terminal region of ARX (1–220) is required for this interaction (Fig 4F). The fact that the ARX (431-564-3XSV40-NLS) failed to interact with BCL9 may partially explain why the transactivation activity of the D164A mutant co-expressed with ARX (431-564-3XSV40-NLS) was not fully recovered to the extent achieved with ARX (WT) (Fig 4C). Taken together, our data suggest that the inter-molecular interaction between ARX, BCL9, and β-catenin is crucial for optimal signaling activity.

While BCL9 is a co-activator of β-catenin and it binds to the N-terminus of β-catenin, there are multiple co-activators and chromatin remodeling proteins that bind to the structurally flexible C-terminal region of β-catenin. Examples include TRRAP, p400, MLL1, SNF2H/ISW1, TIP49 and p300/CBP [16]. Thus, we next tested if ARX can synergistically cooperate with these proteins to increase Wnt/β-catenin signaling activity. Using the TOP-flash reporter assay, we found that P300 synergistically up-regulates Wnt/β-catenin activity with ARX (Fig 5A). A GST-pull-down assay reveals that ARX (WT) and ARX (431-564-3XSV40-NLS) can bind to P300 but not ARX (1–220) or ARX (221–430), providing evidence that the C-terminal region of the ARX physically interacts with P300 (Fig 5B). These two results suggest that ARX and P300 interact with each other both physically and functionally. Taken together, our interaction mapping data support a model where ARX modulates Wnt/β-catenin signaling activity through its C-terminal interaction domain (431–564) that binds to the Armadillo domains of the β-catenin and also to P300, as well as its N-terminal region (1–220) that binds to BCL9 (Fig 5C).

## Discussion

Mutations in *ARX* are associated with a spectrum of neurologic disorders and experimental studies indicate that ARX plays distinct roles in different neural progenitor populations [6–11,24]. To better understand how this one transcription factor participates in these multiple functions, we took an unbiased proteomics approach to identify ARX interacting proteins and found several components of the canonical Wnt pathway expressed in cortical (pallial) progenitor cells of the developing forebrain. We determined that ARX enhances Wnt/β-catenin signaling via its interactions with β-catenin, BCL9, and P300. More specifically, the C-terminal region (431–564) of ARX interacts with armadillo repeats of β-catenin for signal regulation. ARX also interacts with BCL9 and P300 [C-terminal region (431–564) of ARX interacts with BCL9 and N-terminal (1–220) with P300], and these interactions appear to synergistically up-regulate canonical Wnt activity. These results provide new mechanistic insight into the pathogenesis of the phenotypes observed in the *Arx* mutant mouse brain and presumably in the patients with *ARX* mutations, connecting Wnt/β-catenin signaling to the pathophysiology of *ARX* related neurological diseases.

We previously described multiple developmental defects in the cerebral cortex of *Arx*^*-/y*^ mice [8]. The data in the present study suggest that the mis-regulation of canonical Wnt signaling might contribute to the cortical neurogenesis defects observed in *Arx*^*-/y*^ mice [8]. Supporting this prediction is the fact that the neocortex of Arx^*-/y*^ and Wnt/β-catenin mutant mice show similar phenotypes; both resulting in part to a premature cessation of neurogenesis [8,25]. Of interest, in our previously reported microarray data, only a subset of Wnt target genes show expression level changes in *Arx* mutant mice [8], suggesting that ARX may modulate only a subset of Wnt downstream targets rather than function as a general Wnt activator. Our data further suggest that Wnt/β-catenin signal needs to be tightly modulated during cortical neurogenesis. In accordance with this, DISC1, ANK3 and ASPM, whose mutations are associated with schizophrenia, bipolar disorder and autism spectrum disorder, have been shown to regulate Wnt/β-catenin signaling activity during cortical neurogenesis [26–30]. Together, our data along with a growing body of data in the literature indicate that the fine regulation of the Wnt/β-catenin signaling is required for normal neocortical development, and our current study suggests that ARX plays an important role in participating in this fine regulation.

To better understand how ARX regulates Wnt**/**β**-**catenin signaling, we have delineated the inter-molecular domain interactions, required for this transcriptional complex to modulate Wnt/β-catenin signaling. The C-terminal region (431–564) of ARX, near the aristaless domain, was found to be crucial for up-regulating Wnt/β-catenin signaling. Interestingly, within this C-terminal region (431–564), the aristaless domain (529–544) itself, which is highly conserved from invertebrates to vertebrates and known to play a role in transcriptional activation [18], does not appear to be required for up-regulating Wnt/β-catenin signaling. Rather, the putative minimal domain (431–528), which is not conserved in fly but is in vertebrates, appears to be the critical region, given that full-length ARX with multiple mutations in the conserved amino acids of the aristaless domain retains transactivation activity in the Wnt/β-catenin reporter assay (Fig 2). Furthermore, our data with the poly-alanine tract expansion (ARX^E^) mutant that also retains Wnt/β-catenin activation activity, provides further evidence that the normal cortical development in *Arx*^*E*^ mutant mice previously observed by our group and others is likely due to preservation of Wnt/β-catenin signaling activity in dorsal cortical progenitors [11,31,32]. In contrast, the ventral interneuron progenitors, which are not dependent on the same Wnt/β-catenin signaling, are affected in the *ARX*^*E*^ mice [11]. These data are consistent with our hypothesis that ARX has distinct functions in these two different progenitor populations [11]

The data in this report further support an emerging body of evidence that one transcription factor, in this case ARX, has unique roles in different progenitor cells and that these distinguishable functions can be associated with specific neurologic disorders [2,33]. This model has largely been derived from mice engineered to be deficient for *Arx* selectively in pallial or subpallial progenitor neurons [34,35]. These mice exhibit distinct abnormalities that reflect similar phenotypes reported in patients with *ARX* mutations, depending on where *Arx* is deficient [8,24,34–36]. The epilepsy observed in both humans and mice is referable to loss of *Arx* from subpallial progenitor cells (GE derived), which will eventually migrate to the cortex and differentiate into cortical inhibitory interneurons [34,35]. This epileptic phenotype is also observed in mice with an Alanine track expansion in ARX and, interestingly, these mice have a defect specifically in the subpallium as we have shown previously [11]. These mice and patients do not have structural defects in the brain [11,37,38], suggesting that the loss of *Arx* function in the subpallial cells causes epilepsy without gross structural brain defects. In contrast, loss of *Arx* from pallial progenitors results in both structural and behavioral deficits, but not epilepsy [34]. We have identified cell proliferation as one cellular function that contributes to the pathobiology of this projection neuron phenotype [8]. The data in this manuscript support this model.

In summary, beginning with an unbiased proteomics screen to identify ARX interacting proteins, we found components of a transcriptional complex that positively modulate the Wnt/β-catenin signaling pathway in the dorsal, cortical progenitor cells. These data also provide new insights into the pathogenesis of the malformations observed in patients with *ARX* mutations. We anticipate our ongoing studies will identify unique transcriptional pathways in ventral GE progenitors where ARX is known to play a distinct role from the dorsal progenitor cells.

## Supporting Information

S1 Table List of primers used for constructs used in this study. (PDF)

S2 Table List of identified proteins in MASS/SPEC. (XLSX)

S1 Fig *Arx*, *Lrrfip2*, *Bcl9* and *Ctnnb1* show prominent expression in the developing mouse cortex. Sagittal sections of mouse embryonic brain showing mRNA *in situ* hybridization data from the public databases GenePaint (http://www.genepaint.org/) (Arx, Lrrfip2, Bcl9 as E14.5) or Allen Brain Atlas (http://www.brain-map.org/) (Ctnnb1as E15.5).
(TIF)

S2 Fig ARX regulation of Wnt signaling is Wnt3a concentration dependent. The level of luciferase induction by Arx or control in the cells treated with different concentration of rWnt3a is indicated as RLU (relative light unit). rWnt3a, recombinant Wnt3a.
(TIF)

S3 Fig NLS3 is critical for nuclear localization of the ARX. ARX (321–564), which contains nuclear localization sequence 2 (NLS2) and NLS3, is localized to the nucleus, whereas ARX (321–564) with mutated NLS3 (mtNLS3) failed to localize to the nucleus. When NLS from SV40 was added to ARX (321–564)-mtNLS3, nuclear localization of the ARX protein was restored. ARX (431–564) failed to localize to the nucleus but when three copies of the SV40 NLS were attached, this protein was successfully localized to the nucleus.
(TIF)
