# Supplementary material for: Aristaless Related Homeobox (ARX) Interacts with β-Catenin, BCL9, and P300 to Regulate Canonical Wnt Signaling
Source: PLoS One. 2017 Jan 19;12(1):e0170282. doi: 10.1371/journal.pone.0170282 (PMC5245867; doi:10.1371/journal.pone.0170282)
Supplement: S1 Table — (PDF) [file pone.0170282.s001.pdf]

pCIG-Insert-myc	Forward primer (F-5' to 3')<br>Reverse primer (R-5' to 3')
Arx (1-564-myc)	F- CTAGAATTCCACCATGAGCAATCAGTACCAG<br>R- CAACGCGTCTACAGATCTTCTTCAGAAATAAGTTTTTGTTC GCACACCTCCTTCCCCGTG
Arx (1-220)	F- CTAGAATTCCACCATG AGCAATCAGTACCAGGAAGAG<br>R- CAACGCGT CTACAGATCTTCTTCAGAAATAAGTTTTTGTTC CGTGCCACCACCCGCCG
Arx (221-430)	F- CTAGAATTCCACCATG GGCGCCGAGGACGACGAG<br>R- CAACGCGT CTACAGATCTTCTTCAGAAATAAGTTTTTGTTC CGAATCAAGCGCAGGGTGATG
Arx (431-564)	F- CTAGAATTCCACCATG GCCTGGACCGCCGCGGC<br>R- CAACGCGTCTACAGATCTTCTTCAGAAATAAGTTTTTGTTC GCACACCTCCTTCCCCGTG
Arx (1-470)	F- CTAGAATTCCACCATG AGCAATCAGTACCAG<br>R-CAACGCGTCTACAGATCTTCTTCAGAAATAAGTTTTTGTTC TGCTCCTAGAAAAGTGCTCAG
Arx (321-564)	F- CTAGAATTC CACC ATG TCGGAGGAGGGGCTGC<br>R- CAACGCGT CTACAGATCTTCTTCAGAAATAAGTTTTTGTTC GCACACCTCCTTCCCCGTG
Arx (381-564)	F- CTAGAATTCCACCATG CGTCGGGCCAAGTGGCG<br>R- CAACGCGT CTACAGATCTTCTTCAGAAATAAGTTTTTGTTC GCACACCTCCTTCCCCGTG
Arx (321-564-mtNLS3)	F- GTGTGGTTCCAGAACGCTGCGGCCGCGTGGCGCGCGCGGGAGAAGGCTGG<br>R- CCAGCCTTCTCCCGCGCGCGCCACGCGGCCGCAGCGTTCTGGAACCACAC
Arx (321-564-mtNLS3-SV40-NLS)	F- CTAGAATTCCACCATGTCGGAGGAGGGGCTGC<br>R- CTAACGCGTTCATACCTTTCTCTTCTTTTTTGGATC CAGATCTTCTTCAGAAATAAG
Arx (471-564)	F- CTAGAATTCCACCATG GCTGTGTTCCGCCACCCAG<br>R- CAACGCGTCTACAGATCTTCTTCAGAAATAAGTTTTTGTTC GCACACCTCCTTCCCCGTG
Arx (471-564-3xSV40 NLS)	F- CTAGAATTCCACCATG GCTGTGTTCCGCCACCCAG<br>R-CGCACGCGTTCATACCTTTCTCTTCTTTTTTGGATCTACCTTTCTCTTCTTTTTTGGATC TACCTTTCTCTTCTTTTTTGGATC CAGATCTTCTTCAGAAATAAG
Arx (431-564-3xSV40 NLS)	F- CTAGAATTCCACCATG GCCTGGACCGCCGCGGC<br>R-CGCACGCGTTCATACCTTTCTCTTCTTTTTTGGATCTACCTTTCTCTTCTTTTTTGGATC TACCTTTCTCTTCTTTTTTGGATC CAGATCTTCTTCAGAAATAAG
Arx (321-532)	F- CTAGAATTCCACCATG TCGGAGGAGGGGCTGC<br>R- CAACGCGTCTACAGATCTTCTTCAGAAATAAGTTTTTGTTC TGACGCGCGTCTGTCTGCC
Arx (1-564-mtAristaless)	F- TGAGCCTCAGCGCGGCTGCGGCTGCCGCGGCTGCGTCTGCCGCGGCGGTGG<br>R- CCACCGCCGCGGCAGACGCAGCCGCGGCAGCCGCAGCCGCGCTGAGGCTCA
Arx (1-564-Δ471-528)	F- GCTTGACGCGCGTCTTGCTCCTAGAAAAGTGCT<br>R- GCTTGACGCGCGTCTTGCTCCTAGAAAAGTGCT
Arx (Ala Exp)	F- GGCCGCTGCTGCTGCTGCTGCCGC<br>R- GGCCGCGGCAGCAGCAGCAGCAGC
Arx (R332H)	F- CGTGGTGCGGTAGTGCCTCTGTTTGCG<br>R- CGCAAACAGAGGCACTACCGCACCACG
ΔN-β-Cat/Tcf4 (Tcf7L2)	F-TCGAGCTCAAGCTTCG CACC ATG AGGGCTCAGAGGGTCCGAGC<br>R-CGCTCGTCACCAAGTCTTTAGAATGATCTGCAGAAGCTTCTAG
VP16-DBD/ND-Tcf4 (Tcf7L2)	F- TCGAGCTCAAGCTTCG CACC ATGTTGGGGGACGGGGATTC<br>R- CGCTCGTCACCAAGTCTTTAGAATGATCTGCAGAAGCTTCTAG
Lrrfip2-HA	F- TCGAGCTCAAGCTTCG CACC ATGGGGACTCCTGGT<br>R- TAGAAGCTTCTGCAGACTAAGCGTAATCTGGAACATCGTATGGG TACTGCTGGGCTAGAAGGGCTG
Bcl9-HA	F- TCGAGCTCAAGCTTCG CACC ATGCATCCCAGTAACCCTAAAG<br>R- TAGAAGCTTCTGCAGACTAAGCGTAATCTGGAACATCGTATGGG TAAAACATCATGTTTCCTGGGTTAC
pCIG-Insert	
β-Catenin (1-781)	F- CTAGAATTCCACCATGGCTACTCAAGCTGACCTGATGG<br>R- CAACGCGTTCACAGGTCAGTATCAAACCAGG

pEBG-GST-Insert	
Arx (2-564)	F- GATCTGGTTCCGCGTGGAAGCAATCAGTACCAGGAAGAG<br>R- CTCACTCTAGAGTCGCTCAGCACACCTCCTTCCCCGTG
Arx (2-220)	F- GATCTGGTTCCGCGTGGAAGCAATCAGTACCAGGAAGAG<br>R- CTCACTCTAGAGTCGCTCACGTGCCACCACCCGCCG
Arx (221-430)	F- GATCTGGTTCCGCGTGGAGGCGCCGAGGACGACGAG<br>R- CTCACTCTAGAGTCGCTCACGAATCAAGCGCAGGGTGATG
Arx (431-564-3xSV40 NLS)	F- GATCTGGTTCCGCGTGGCCTGGACCGCCGCGGC<br>R- CTCACTCTAGAGTCGCCTTCTGCAGACGCGTTCATACC
β-Catenin (2-781)	F- GATCTGGTTCCGCGTGGAGCTACTCAAGCTGACCTG<br>R- CTCACTCTAGAGTCGCTCACAGGTCAGTATCAAACCAGGC
β-Catenin (2-781-S33Y)	F- GAATGGATTCCATAATCCAAGTAAGACTGCTGCTGC<br>R- GCAGCAGCAGTCTTACTTGGATTATGGAATCCATTC
β-Catenin (90-781)	F- CTAGGATCCAGGGCTCAGAGGGTCCGAGC<br>R- CTCACTCTAGAGTCGCTCACAGGTCAGTATCAAACCAGGC
β-Catenin (90-694)	F- CTAGGATCCAGGGCTCAGAGGGTCCGAGC<br>R- CAGCGGCCGCTCATGCAGTCTCATTCCAAGCCATTG
β-Catenin (695-781)	F- CTAGGATCCGATCTTGGACTGGACATTGGTG<br>R- CAGCGGCCGCTCACAGGTCAGTATCAAACCAGGC
β-Catenin (90-530)	F- CTAGGATCCAGGGCTCAGAGGGTCCGAGC<br>R- CAGCGGCCGCTCACTGTTCCCGCAAAGGCGCATG
β-Catenin (531-694)	F- CTAGGATCCGGTGCTATTCCACGACTAGTTC<br>R- CAGCGGCCGCTCATGCAGTCTCATTCCAAGCCATTG
Bcl9	F- GATCTGGTTCCGCGTGGACATCCCAGTAACCCTAAAGTG<br>R- CTCACTCTAGAGTCGCTCAAAACATCATGTTTCCTGGGTTAC
pGal4-DBD-Insert	
β-Catenin (90-781)	F- CTACAATTGAGGGCTCAGAGGGTCCGAGC<br>R- ACTCTAGATTACAGGTCAGTATCAAACCAGG
β-Catenin (90-781-D164A)	F- CTTTATTAACTACCACCTGGGCCTCATCGTTTAGCAGTTTT<br>R- AAAACTGCTAAACGATGAGGCCCAGGTGGTAGTTAATAAAG
β-Catenin (90-694)	F- TATGATCATTACTTATCTAGATT ATGCAGTCTCATTCCAAGCCATTGG<br>R- CCAATGGCTTGGAATGAGACTGCATAATCTAGATAAGTAATGATCATA
pcDNA3.1-Insert	
Bcl9-HA	F- TCGAGCTCAAGCTTCGCACCATGCATCCCAGTAACCCTAAAG<br>R- TAGAAGCTTCTGCAGACTAAGCGTAATCTGGAACATCGTATGGG<br>TAAAACATCATGTTTCCTGGGTTAC
