# Supplementary figures and images for: Aristaless Related Homeobox (ARX) Interacts with β-Catenin, BCL9, and P300 to Regulate Canonical Wnt Signaling
Source: PLoS One. 2017 Jan 19;12(1):e0170282. doi: 10.1371/journal.pone.0170282 (PMC5245867; doi:10.1371/journal.pone.0170282)

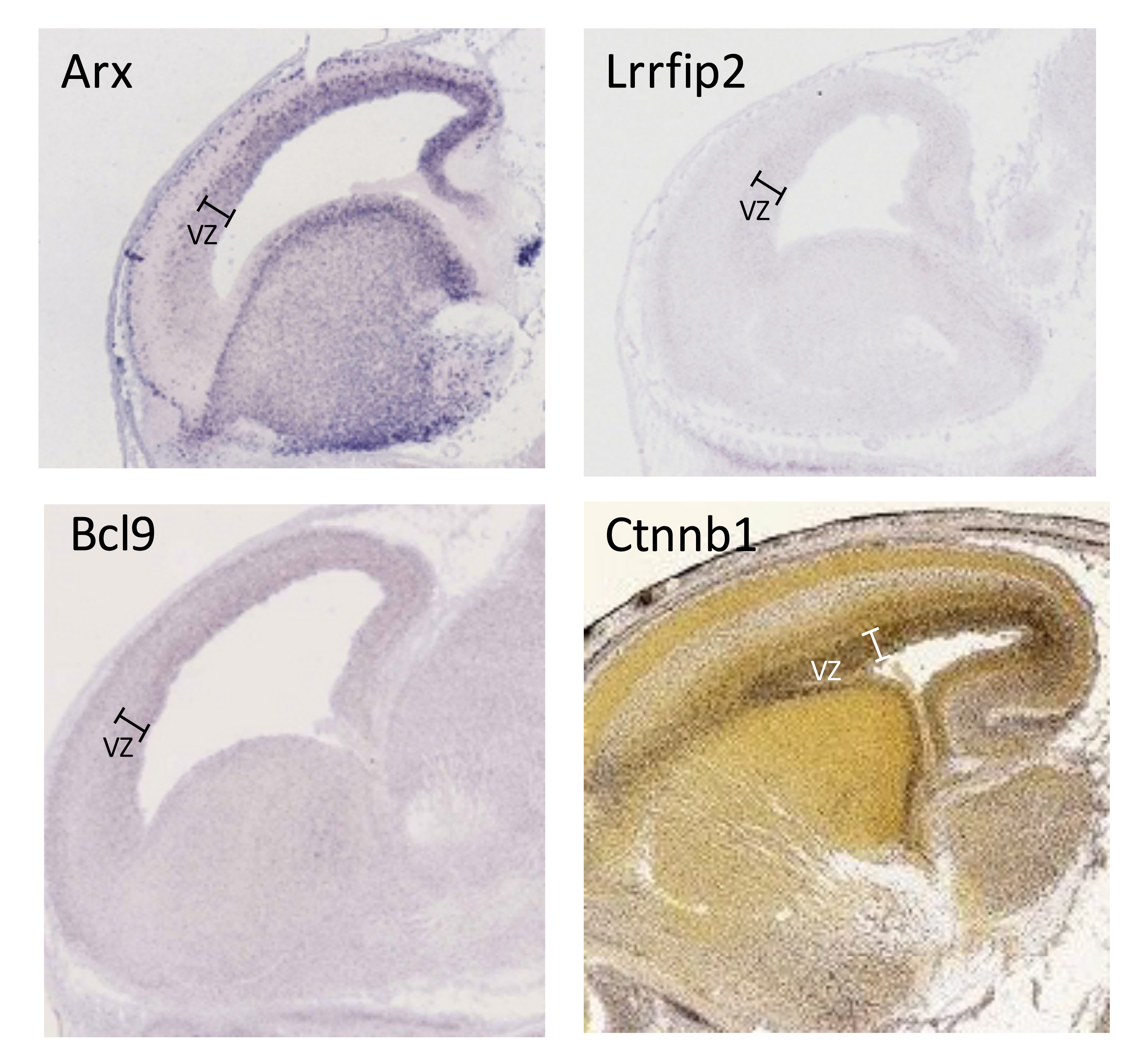

Supplement: S1 Fig — Sagittal sections of mouse embryonic brain showing mRNA in situ hybridization data from the public databases GenePaint (http://www.genepaint.org/) (Arx, Lrrfip2, Bcl9 as E14.5) or Allen Brain Atlas (http://www.brain-map.org/) (Ctnnb1as E15.5). (TIF) [file pone.0170282.s003.tif]

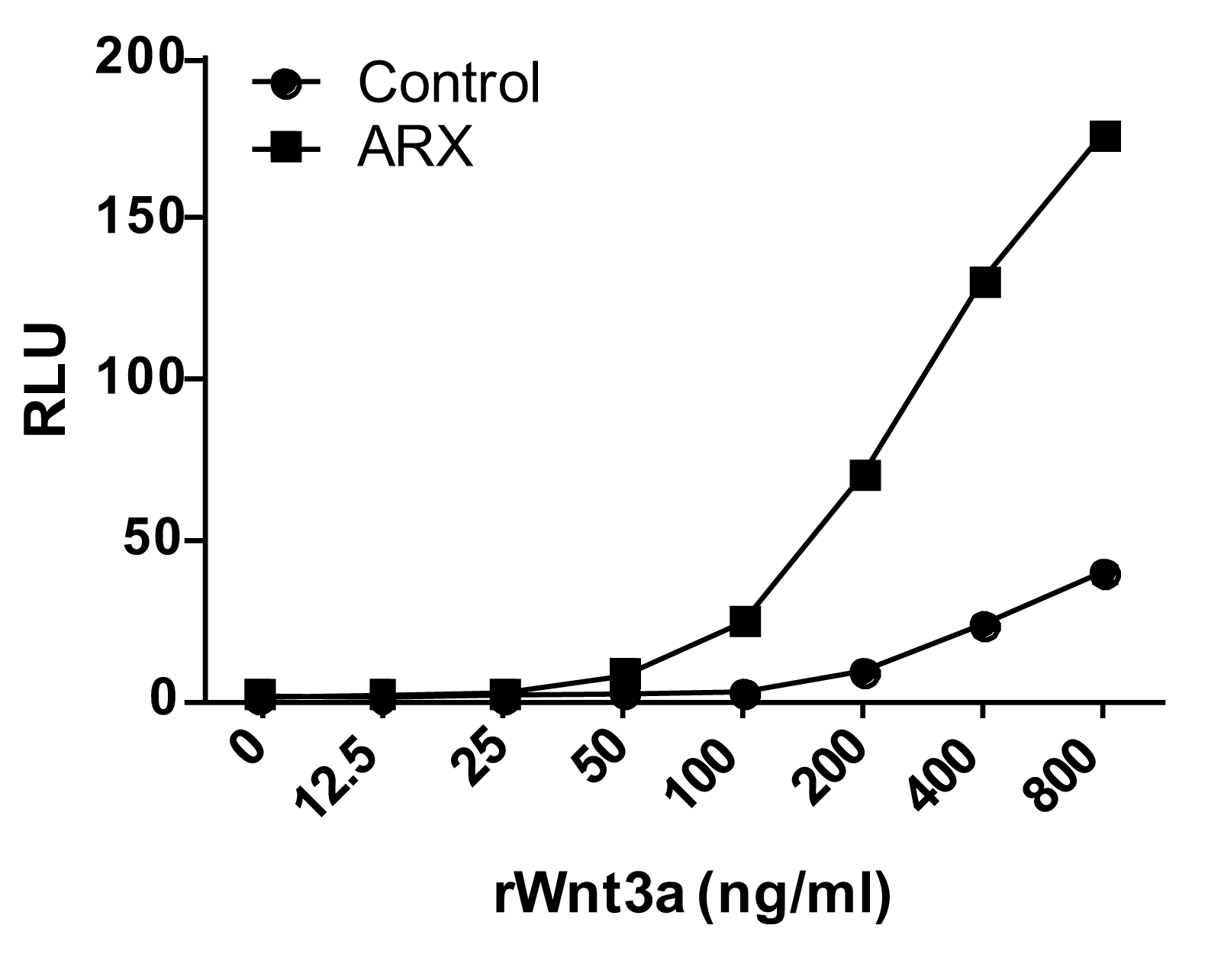

Supplement: S2 Fig — The level of luciferase induction by Arx or control in the cells treated with different concentration of rWnt3a is indicated as RLU (relative light unit). rWnt3a, recombinant Wnt3a. (TIF) [file pone.0170282.s004.tif]

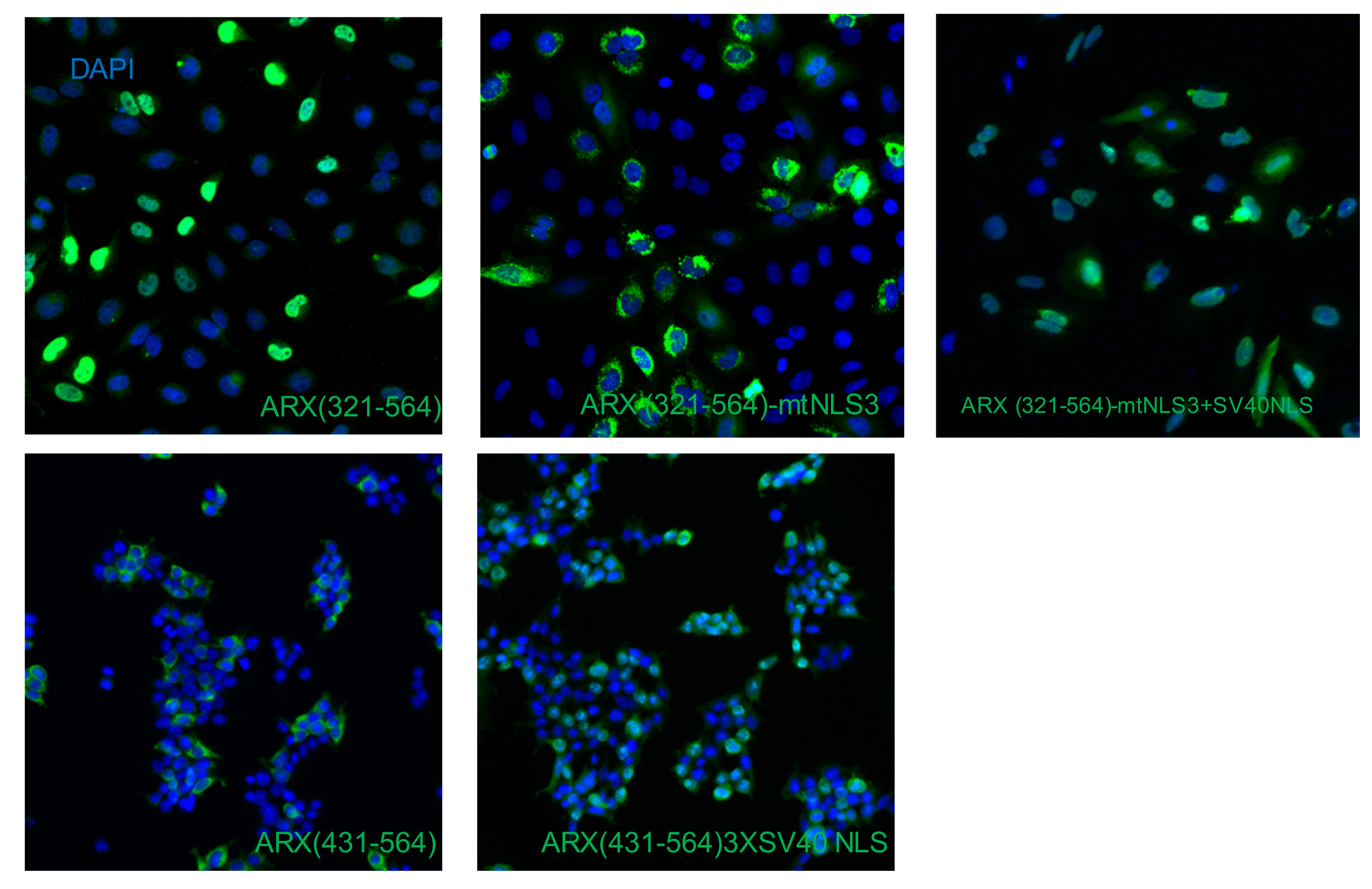

Supplement: S3 Fig — ARX (321–564), which contains nuclear localization sequence 2 (NLS2) and NLS3, is localized to the nucleus, whereas ARX (321–564) with mutated NLS3 (mtNLS3) failed to localize to the nucleus. When NLS from SV40 was added to ARX (321–564)-mtNLS3, nuclear localization of the ARX protein was restored. ARX (431–564) failed to localize to the nucleus but when three copies of the SV40 NLS were attached, this protein was successfully localized to the nucleus. (TIF) [file pone.0170282.s005.tif]
